# Combination of an aurora kinase inhibitor and the ABL tyrosine kinase inhibitor asciminib against ABL inhibitor-resistant CML cells

**DOI:** 10.1007/s12032-024-02394-6

**Published:** 2024-05-07

**Authors:** Seiichi Okabe, Mitsuru Moriyama, Akihiko Gotoh

**Affiliations:** Department of Hematology, Tokyo Medical University, 6-7-1 Nishi-Shinjuku, Shinjuku-Ku, Tokyo, 160-0023 Japan

**Keywords:** Chronic myeloid leukemia, BCR::ABL1, Aurora kinase inhibitor, Asciminib, ABL tyrosine kinase inhibitor resistance

## Abstract

The development of BCR::ABL1-targeting tyrosine kinase inhibitors (TKIs) has improved the prognosis of patients with chronic myeloid leukemia (CML). However, resistance to ABL TKIs can develop in CML patients due to BCR::ABL1 point mutations and CML leukemia stem cell (LSC). Aurora kinases are essential kinases for cell division and regulate mitosis, especially the process of chromosomal segregation. Aurora kinase members also promote cancer cell survival and proliferation. This study analyzed whether aurora kinases were regulated in the progression of CML. It also evaluated the efficacy of the ABL TKI asciminib and the aurora kinase inhibitor LY3295668. The expressions of *AURKA* and *AURKB* were higher in the CML cells compared with normal cells using a public database (GSE100026). Asciminib or LY3295668 alone inhibited CML cells after 72 h, and cellular cytotoxicity was increased. The combined use of Asciminib and LY3295668 increased superior efficacy compared with either drug alone. Colony formation was reduced by cotreatment with asciminib and LY3295668. In the cell-cycle analyses, LY3295668 induced G2/M arrest. Cell populations in the sub-G1 phase were observed when cotreating with asciminib and LY3295668. The combination treatment also changed the mitochondrial membrane potential. In addition, AURKA shRNA transfectant cells had increased asciminib sensitivity. Combining asciminib and aurora kinase inhibition enhanced the efficacy and is proposed as a new therapeutic option for patients with CML. These findings have clinical implications for a potential novel therapeutic strategy for CML patients.

## Introductions

Chronic myeloid leukemia (CML) is a kind of cancer that results from the uncontrolled growth of myeloid cells in the bone marrow [1]. This condition is classified into three stages: chronic phase (CP), accelerated phase (AP), and blastic phase (BC) [2]. The BCR::ABL fusion gene is a genetic alteration that leads to the formation of the BCR::ABL tyrosine kinase protein, which is a driver of CML [1]. The development of tyrosine kinase inhibitors (TKIs) targeting the BCR::ABL1 fusion gene has improved the prognosis of CML patients [3]. However, it is estimated that more than 25% of CML patients will switch to a different TKI at least once in their lifetime due to TKI intolerance or resistance [4]. Mutations within the kinase domain of BCR::ABL1 are the most extensively studied mechanisms of TKI resistance in CML; however, these mutations fail to explain 20–40% of resistant cases [4]. In these instances, the activation of alternative BCR::ABL1-independent survival pathways has been mechanistically implicated. In the bone marrow, CML leukemia stem cells (LSCs) reside. In humans, a CML LSC is operationally defined as a leukemia cell resistant to ABL TKIs after being transferred to immune-deficient mice and passing one or more in vitro or in vivo assays [5]. In clinical practice, the four commercially available TKIs for the frontline treatment of CML are imatinib, dasatinib, nilotinib, and bosutinib [6]. The regulatory approval for the treatment of CML was granted to asciminib, a fourth-generation TKI, in 2021. [7]. Asciminib operates through a unique mechanism that involves binding to the myristoyl pocket of ABL1 and allosterically inhibiting the overactive kinase activity. Specifically, asciminib was used to treat CP CML patients previously exposed to two or more TKIs or had the T315I mutation [7].

Aurora kinases are a group of serine/threonine kinases that possess a highly conserved catalytic domain with auto-phosphorylating sites [8]. This family consists of three members: Aurora kinase A (AURKA), Aurora kinase B (AURKB), and Aurora kinase C (AURKC). Research suggests that Aurora kinase members have oncogenic properties due to their involvement in mitotic activity, promoting cancer cell survival and proliferation. AURKA, in particular, is a crucial mediator for the maturation and separation of centrosomes and the formation of the mitotic spindle during mitosis [9].

AURKA is a serine/threonine kinase that plays a crucial role in several fundamental biological processes, such as the G2/M phase transition, mitotic spindle formation, and DNA replication and is primarily located in the centrosome and the microtubule area near the centrosome [10]. Recent research has identified it as a synthetic lethal partner of multiple tumor suppressors.

Because of its important role in cell division, AURKA is often overexpressed in tumors and has been linked to poor clinical outcomes, making it an appealing target for cancer therapies [8]. Previous research has shown that BCR::ABL1 tyrosine kinase-dependent hyper-activation of the AURKA-Polo-like kinase 1 (PLK1)-FOXM1 axis is associated with imatinib resistance [11].

Currently, there is ongoing research to determine the effects of ABL TKI resistance. This study aimed to assess the efficacy of an aurora kinase inhibitor in CML cell lines that exhibit resistance to nilotinib and ponatinib. Additionally, it investigated whether combining asciminib with an aurora kinase inhibitor could enhance the cytotoxic effects in CML cell lines.

## Materials and methods

### Reagents

LY3295668 (AK-01), a potent aurora kinase inhibitor, was obtained from Selleck Chemicals (Houston, TX, USA), and asciminib (ABL001), a highly selective BCR::ABL inhibitor, was obtained from ActiveBiochem (Maplewood, NJ). Both inhibitors were dissolved in dimethyl sulfoxide (DMSO) to prepare a stock solution at a final concentration of 10 mM and stored at -20 °C in small aliquots. All other chemicals were obtained from Merck KGaA (Darmstadt, Germany).

### Cell line and Cell Culture

The CML cell line, K562, was purchased from the American Type Culture Collection (Manassas, Virginia, USA). Additionally, previously established ABL TKI-resistant K562 cell lines, including the nilotinib-resistant K562 NR and ponatinib-resistant K562 PR variants, were utilized. Ba/F3 BCR-ABL and BCR-ABL point mutant cells with the T315I mutation have been previously described [12]. These cell lines were cultured in Roswell Park Memorial Institute 1640 (RPMI 1640) medium supplemented with 10% fetal bovine serum at 37 °C with 5% CO2 and were passaged for less than 6 months.

### Data collection and processing

To determine the differentially expressed and spliced transcripts in CML, the mRNA profiles of peripheral blood mononuclear cells from five patients with BC CML and five healthy volunteers were previously generated by deep sequencing using an Illumina NextSeq 500 (GSE100026). This database was downloaded from the Gene Expression Omnibus (GEO https://www.ncbi.nlm.nih.gov/geo/query) [13]. EdgeR, an R package for examining differential expression of RNA-Seq count data, was used according to the user’s guide. Visualization of the RNA-Seq data was analyzed by the open bioinformatics web tool, RaNA-Seq (available on http://ranaseq.eu) [14].

### Cell proliferation assay

CML cells were subjected to treatment with asciminib and/or LY3295668 for 72 h, following which cell proliferation assays were performed using a cell counting kit-8 (Dojindo Laboratories, Mashikimachi, Kumamoto, Japan). The absorbance was then measured at a wavelength of 450 nm using an EnSpire Multimode Plate Reader (PerkinElmer, Waltham, MA, USA).

### Caspase 3/7 activity

To evaluate the caspase activity, a Caspase Glo® 3/7 assay kit from Promega was utilized (Madison, WI, USA), and the manufacturer’s guidelines were followed. The luminescence of each sample was measured after a 48-h incubation period with the specified concentrations of asciminib and/or LY3295668 using an EnSpire Multimode Plate Reader.

### Cytotoxicity assay

Cytotoxicity analysis was conducted on CML cells that were exposed to various concentrations of asciminib and/or LY3295668 for 48 h. The cytotoxicity was assessed by measuring the release of lactate dehydrogenase (LDH) using a Cytotoxicity LDH Assay Kit-WST from Dojindo Laboratories. The absorbance at 490 nm, which indicates the amount of LDH released from dead cells, was measured using an EnSpire Multimode Plate Reader.

### Quantitative real-time reverse transcription-polymerase chain reaction analysis

Quantitative real-time reverse transcription polymerase chain reaction (RT-qPCR) was performed on myeloma cells. Total RNA was extracted using an RNAqueous-4PCR Kit from Life Technologies Japan KK (Minato-ku, Tokyo, Japan). A First-Strand cDNA Synthesis Kit from OriGene Technologies (Rockville, MD, USA) was used to perform the reverse transcription. The RT-qPCR was carried out using a Roche Light Cycler 2.0 detection system, and specific primers for AURKA and β-actin were purchased from Takara Bio Inc.(Otsu, Shiga, Japan). A SYBR Green PCR Kit from Roche was used to quantify the expression of specific genes following the manufacturer’s protocol.

### Short-hairpin RNA transfection

A short-hairpin RNA (shRNA) mammalian AURKA gene expression lentiviral vector was utilized for the transfection. The control shRNA vector was obtained from VectorBuilder Japan, Inc. (Yokohama, Kagawa, Japan). K562 cells were cultured in a six-well culture dish with RPMI 1640 medium supplemented with 8 µg/mL Polybrene (hexadimethrine bromide) (Merck KGaA) for 24 h. The cells were subsequently infected with the lentiviral vectors following the manufacturer’s instructions. After a 24-h incubation, the medium was replaced, and AURKA expression was assessed through an RT-qPCR.

### Colony formation assay

Colony formation assays were carried out using MethoCult® Express (Catalog #04437; STEMCELL Technologies, Vancouver, BC, Canada), a methylcellulose-based growth medium, as previously described [15]. In brief, 10^2^ cells were seeded onto MethoCult™ Express with the specified concentrations of asciminib and/or LY3295668. The plates were then incubated at 37 °C and 5% CO2 for 7 days. The colony counts were determined, and the images were captured using an EVOS™ FL Digital Inverted Fluorescence Microscope (Thermo Fisher Scientific Inc., Waltham, MA, USA). The experiments were repeated three times, and the mean and standard error were calculated and displayed.

### Reactive oxygen species (ROS) assay

The effect of asciminib and/or LY3295668 on the activity of reactive oxygen species (ROS) in cells cultured in RPMI medium was assessed by incubating cells for 24 h. To assess ROS activity, a ROS Assay Kit-Highly Sensitive DCFH-DA kit (Dojindo) was utilized following the manufacturer’s protocol, and an EnSpire Multimode Plate Reader was used for the measurements.

### β-Galactosidase staining

Senescence-associated β-Galactosidase (SA-β-gal) staining was employed in CML cells to measure senescence. The qualitative assessment of SA-β-gal activity was performed using an SA-β-gal Staining Kit (Cell Signaling Technology, Inc., Danvers, MA, USA) according to the manufacturer’s instructions. The percentage of senescent cells was determined by counting the number of stained cells from the total number of cells, which was done using a microscope (Olympus Corporation, Shinjuku-ku, Tokyo, Japan).

### Cell cycle analysis

The cell cycle phases were determined using a BD Cycletest™ Plus DNA Reagent Kit (Becton–Dickinson, Mountain View, CA, USA), following the manufacturer’s guidelines. Specifically, CML cells were grown in the presence of asciminib (10 nM) and/or LY3295668 (100 nM) for 24 h. The DNA content distribution was then measured using a BD FACSVerse™ Flow Cytometer (Becton–Dickinson) and analyzed using BD FACSuite software.

### Statistical analyses

Prism 10 software (GraphPad Software, San Diego, CA, USA) was used to analyze all the presented data. Two-tailed Student’s t-tests were used to test for statistical significance. If one of the groups in the study was considered the control group, data was analyzed using a Dunnett’s test as the post-hoc test following an ANOVA. When comparing three or more samples, data were analyzed using a one-way ANOVA with Turkey post hoc comparison tests with an alpha of 0.05. All experiments were done at least three times (*n* ≥ 3). Significance was expressed as the *p*-value where: *p < 0.05, **p < 0.01, ***p < 0.001, and ****p < 0.0001.

## Result

### Gene expressions of Aurora kinases and activity of the aurora kinase inhibitor in CML cell lines

**Fig. 1:**
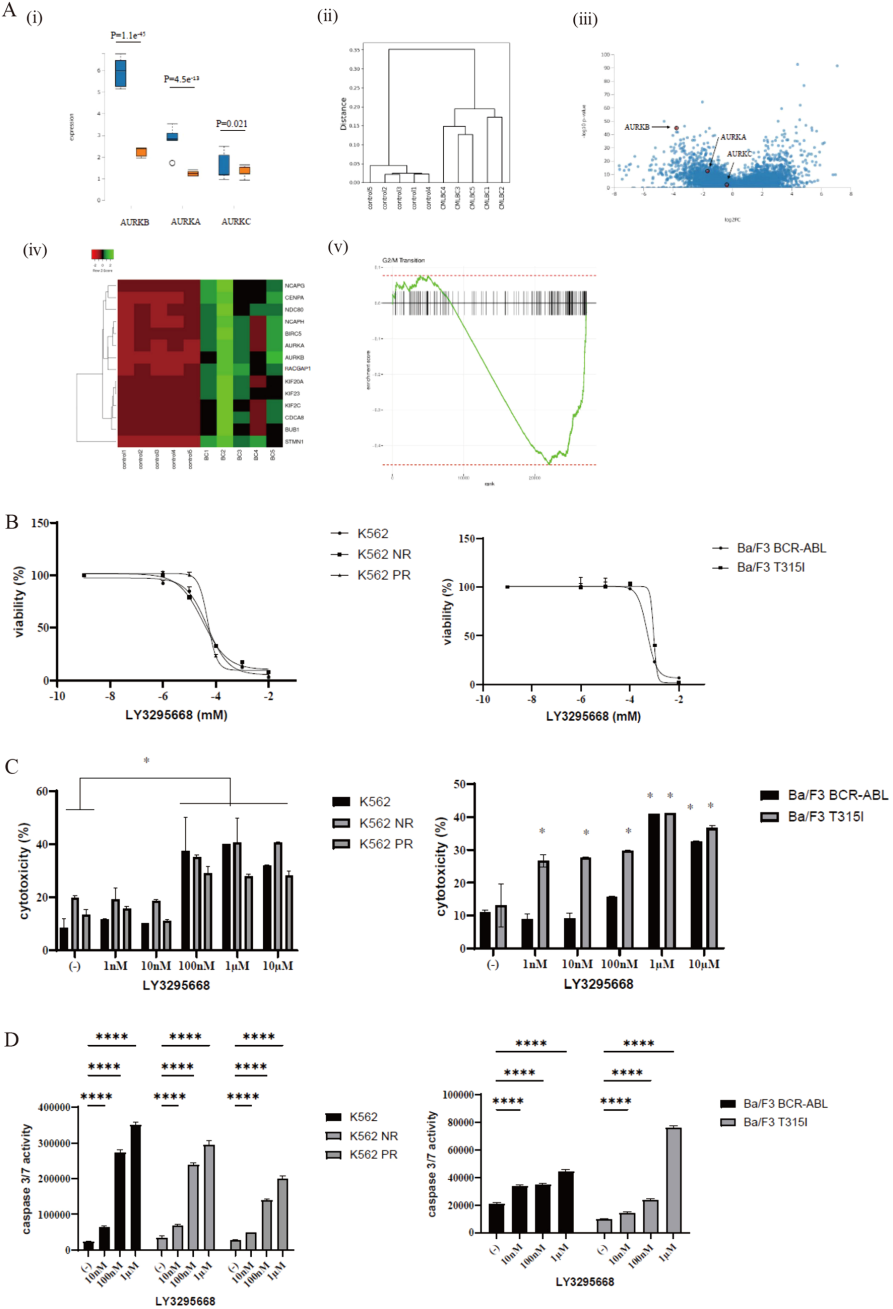
Gene expressions of *AURKA* and *AURKB* in the CML patients and activity of LY3295668 against CML cell lines. **A** Gene expressions of *AURKA* and *AURKB*. To validate the *AURKA* and *AURKB* genes identified by the GEO data (GSE100026), five normal patient samples (control) were compared with five BC samples. Gene expressions of AURKA, AURKB, and AURKC (i). Dendrogram of leukemia patients and control samples (ii), volcano plots (iii), AURKB-related clusters (iv), and gene-set enrichment (v) were analyzed using the open bioinformatics web tool, RaNA-Seq (available on http://ranaseq.eu). **B** CML cell lines (K562, K562 NR, K562 PR, Ba/F3 BCR-ABL, and Ba/F3 T315I) were cultured in RPMI 1640 medium supplemented with 10% FBS with the indicated concentration of LY3295668 for 72 h. Cell viability was evaluated using a cell counting kit-8. **C** CML cell lines (K562, K562 NR, K562 PR, Ba/F3 BCR-ABL, and Ba/F3 T315I) were treated with LY3295668 for 48 h. The cytotoxicity was analyzed using a Cytotoxicity LDH Assay kit (^*^p < 0.05 vs. the control). **D** CML cell lines (K562, K562 NR, K562 PR, Ba/F3 BCR-ABL, and Ba/F3 T315I) were cultured with the indicated concentrations of LY3295668 for 48 h. Caspase 3/7 activity was evaluated (****p < 0.0001 vs. the control)

Aurora kinase amplification is common in various cancer cells due to its central role in cell division. To investigate this further, the GEO database was utilized to analyze the gene expressions of aurora kinases. AURKA and AURKB are genes that encode Aurora kinases, and the analysis revealed that the expressions of these genes were increased in BP CML BP patients compared with normal control patients (Fig. 1A). A dendrogram of leukemia patients clustered by hierarchical clustering in the BC CML and normal cell population showed that AURKA and AURKB were increased in BP CML patients compared with normal patients. Volcano plots of the differentially expressed genes revealed that AURKB was particularly upregulated in the BC CML samples. AURKB-related clusters and patterns of genes were also analyzed, and it was found that they were highly upregulated in the BC CML samples. These findings were previously reported in a study that compared global gene expressions between rigorously defined stem and progenitor cells from BC CML patients and similar populations isolated from normal volunteers (GSE100026) [13]. This study uncovered that the expressions of AURKA and AURKB were elevated in BP CML patients compared with the normal control patients (Fig. 1A).

Additionally, a dendrogram was generated using hierarchical clustering to group leukemia patients in the BC CML and normal control populations. Subsequently, the efficacy of the aurora kinase inhibitor, LY3295668, was evaluated. This study indicated that LY3295668 inhibited the proliferation of all CML cell lines, including those that were resistant to ABL TKIs, such as ABL TKI-resistant K562 cells and Ba/F3 T315I cells, in a dose-dependent manner (Fig. 1B). Additionally, the cytotoxicity of LY3295668 was assessed. The cells were incubated with varying concentrations of LY3295668 for 72 h, and the results showed that LY3295668 induced cytotoxicity in a dose-dependent manner (Fig. 1C). Moreover, asciminib increased caspase 3/7 activity in a dose-dependent manner, widely recognized as a reliable indicator of cell apoptosis (Fig. 1D).

### The activity of asciminib combined with LY3295668 in CML cells

**Fig. 2:**
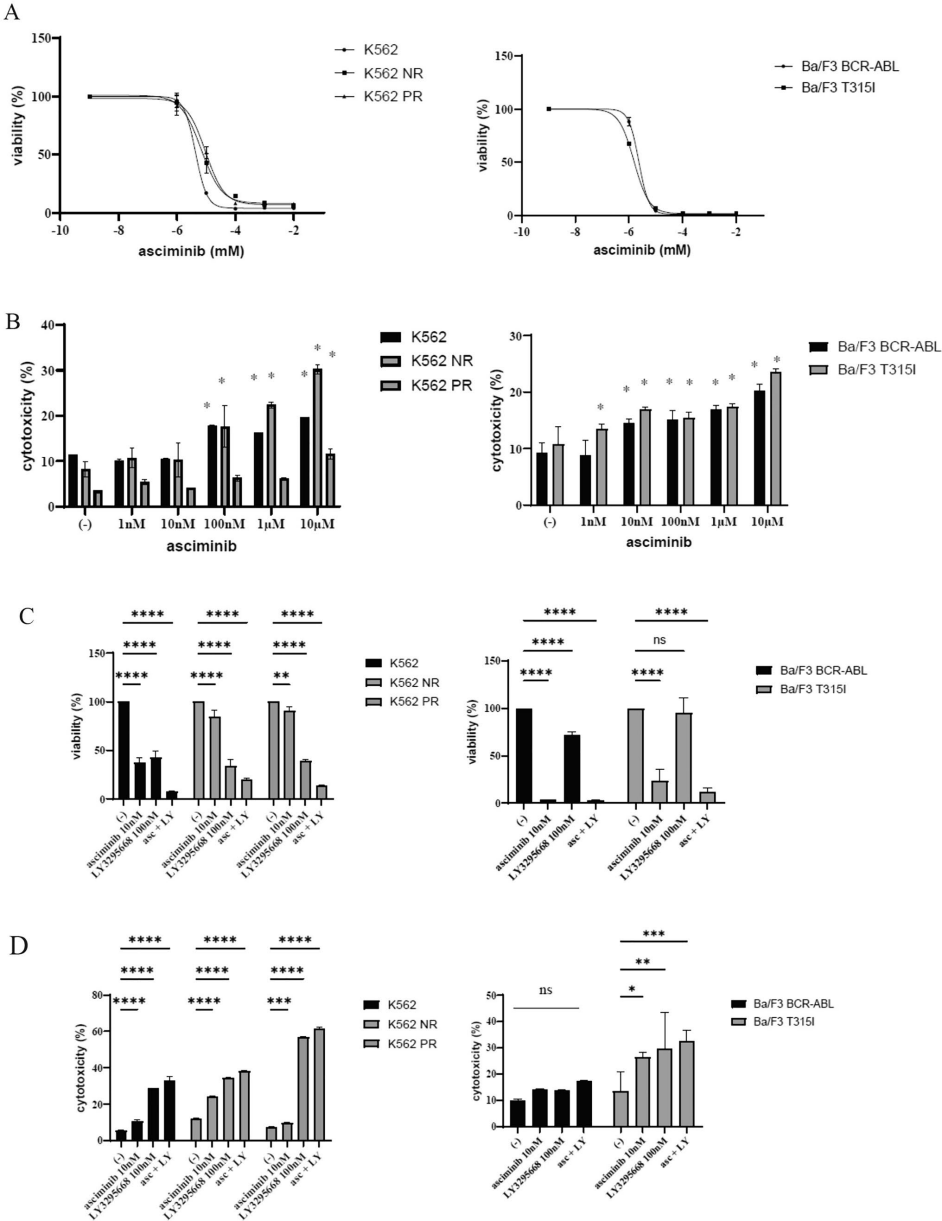
Effects of asciminb and the co-treatment of asciminib and LY3295668 on the CML cell lines. **A** CML cell lines (K562, K562 NR, K562 PR, Ba/F3 BCR-ABL, and Ba/F3 T315I) were cultured in RPMI 1640 medium with the indicated concentration of asciminib for 72 h. Cell viability was evaluated using a cell counting kit-8. **B** CML cell lines (K562, K562 NR, K562 PR, Ba/F3 BCR-ABL, and Ba/F3 T315I) were treated with asciminib for 48 h. The cytotoxicity was analyzed using a Cytotoxicity LDH Assay kit (^*^p < 0.05 vs. the control). **C** CML cell lines (K562, K562 NR, K562 PR, Ba/F3 BCR-ABL, and Ba/F3 T315I) were incubated with asciminib and/or LY3295668 for 72 h. Cell viability was evaluated. Significance is expressed as *p < 0.05, **p < 0.01, ***p < 0.001, ****p < 0.0001, and ns: not significant vs. the control. **D** CML cell lines (K562, K562 NR, K562 PR, Ba/F3 BCR-ABL, and Ba/F3 T315I) were incubated with asciminib and/or LY3295668 for 48 h. Cytotoxicity was evaluated. Significance is expressed as *p < 0.05, **p < 0.01, ***p < 0.001, ****p < 0.0001, and ns: not significant vs. the control

Asciminib is a Specifically Targeting the ABL Myristoyl Pocket (STAMP) inhibitor that has been approved for the treatment of CP CML in patients with prior exposure to at least two TKIs or the presence of the T315I mutation. To assess the activity of asciminib, a CML cell line that exhibited resistance to ABL TKIs was utilized. The cells were incubated with asciminib at various concentrations for 72 h, and cell proliferation was evaluated. The results demonstrated that asciminib effectively inhibited the proliferation of CML cells (Fig. 2A) and induced cytotoxicity in a dose-dependent manner (Fig. 2B). The combined effects of asciminib and LY3295668, another TKI, on cell growth and cytotoxicity were examined. The cells were exposed to asciminib and/or LY3295668 for 72 h, and it was found that the co-treatment with both drugs increased cell growth inhibition compared with each drug alone (Fig. 2C). Co-treatment with asciminib and LY3295668 also led to increased cytotoxicity (Fig. 2D).

### Asciminib and LY3295668 induce cell death in CML cell lines

**Fig. 3:**
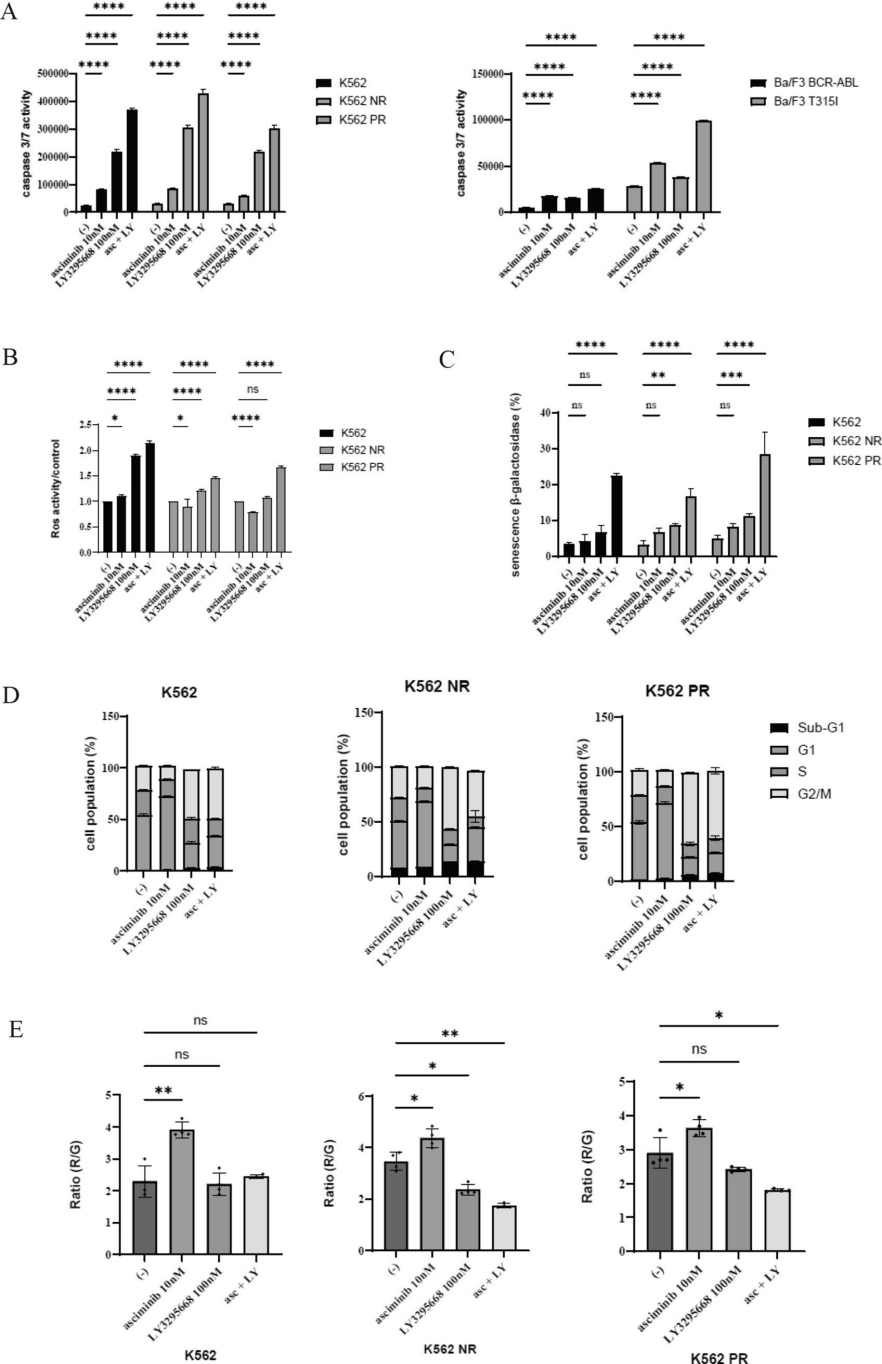
Activity of asciminib and LY3295668 on the CML cell lines. **A** CML cell lines (K562, K562 NR, K562 PR, Ba/F3 BCR-ABL, and Ba/F3 T315I) were treated with asciminib and/or LY3295668 for 48 h. Caspase 3/7 activity was evaluated (****p < 0.0001 vs. the control). **B** CML cell lines (K562, K562 NR, and K562 PR) were treated with asciminib and/or LY3295668 for 48 h. According to the manufacturer’s protocol, ROS activity was analyzed using a ROS Assay Kit-Highly Sensitive DCFH-DA. Significance was expressed as *p < 0.05, ****p < 0.0001, and ns: not significant vs. the control. **C** CML cell lines (K562, K562 NR, and K562 PR) were treated with asciminib and/or LY3295668 for 48 h. A senescence β-Galactosidase Staining Kit was used for the SA-β-gal staining. Significance is expressed as **p < 0.01, ***p < 0.001, ****p < 0.0001, and ns: not significant vs. the control. **D** CML cell lines (K562, K562 NR, and K562 PR) were treated with asciminib and/or LY3295668 for 24 h. Cell cycle phase profiling was determined using a BD Cycletest™ Plus DNA Reagent Kit. A representative histogram for each condition is illustrated. **E** CML cell lines (K562, K562 NR, and K562 PR) were treated with asciminib and/or LY3295668 for 48 h. The MMP was analyzed using a Mitochondria Staining Kit. Significance is expressed as *p < 0.05, **p < 0.01, and ns: not significant vs. the control

To evaluate the effects of the co-treatment with asciminib and LY3295668, CML cell lines were incubated with the specified concentrations of each drug for 48 h. The results indicate that caspase 3/7 activity was increased with the co-treatment compared with either drug alone (Fig. 3A). Elevated levels of ROS have been observed in a wide range of cancers [16], and oxidative stress (OS) has been linked to the development of leukemia. Therefore, ROS activity was examined, and it was found that asciminib and LY3295668 increased Ros activity (Fig. 3B). Replicative senescence, which restricts the proliferation of somatic cells in culture and may reflect cellular aging in vivo, is a widely used biomarker for aging cells. The number of SA-beta-gal positive cells was increased with asciminib and LY3295668 treatment (Fig. 3C). Since CML cell growth was decreased, a cell cycle analysis using flow cytometry was performed. LY3295668 induced G2/M cell cycle arrest, and combining asciminib and LY3295668 increased the sub-G1 population (Fig. 3D). Furthermore, mitochondria membrane potential (MMP), which is an indicator of mitochondrial activity, was measured, and it was observed that asciminib and LY3295668 co-treatment decreased the MMP, particularly in the ABL TKI-resistant cells, but not in the parental cell line, K562 (Fig. 3E).

### Co-treatment with asciminib and LY3295668 impaired colony formation

**Fig. 4:**
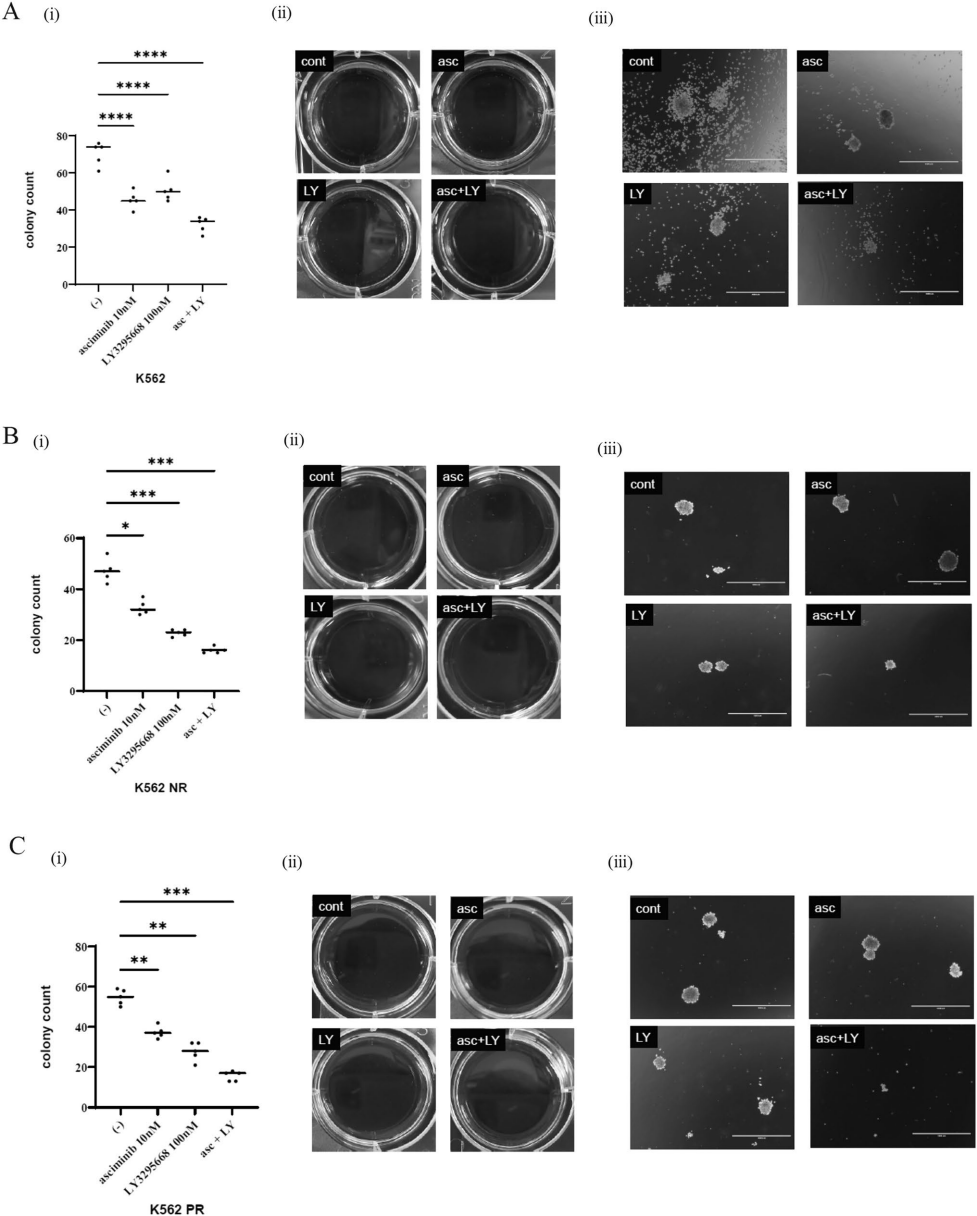
Cell viability was measured using a colony-forming assay according to the treatment with a single reagent or combination of asciminib plus LY3295668 in CML cell lines K562 (**A**), K562 NR (**B**), and K562 PR (**C**) cells were treated with 10 nM asciminib and/or 100 nM LY3295668 for 7 days. The colonies per dish were photographed and counted using a digital camera and an EVOS™ FL Digital Inverted Fluorescence Microscope. The number of colonies detected for 7 days (i), photographs of colonies taken using a digital camera (ii), and a magnified image (4X) of a colony on EVOS™ FL Digital Inverted Fluorescence Microscope (iii). The colony formation and representative images from three independent sets of experiments. Scale bar: 1,000 μm. The results represent three independent experiments. Significance is expressed as *p < 0.05, **p < 0.01, ***p < 0.001, and ****p < 0.0001 vs. the control

The colony formation assay, widely regarded as the benchmark for assessing the effects of cytotoxic agents on cancer cells in vitro, was employed to evaluate the anticancer effects of asciminib and LY3295668. Specifically, the long-term effects of asciminib or LY3295668, as well as their combined effects, were analyzed. The results revealed a significant reduction in the colony count when asciminib and LY3295668 were administered in combination (Fig. 4A). The bright-field images corroborated these findings, demonstrating that the co-treatment with asciminib and LY3295668 reduced colony formation compared with each drug administered alone. Moreover, this study shows that nilotinib-resistant K562 cells and ponatinib-resistant K562 cells were also inhibited by asciminib combined with LY3295668 (Fig. 4B, C).

### Knockdown of AURKA increased asciminib sensitivity in K562 cells

**Fig. 5:**
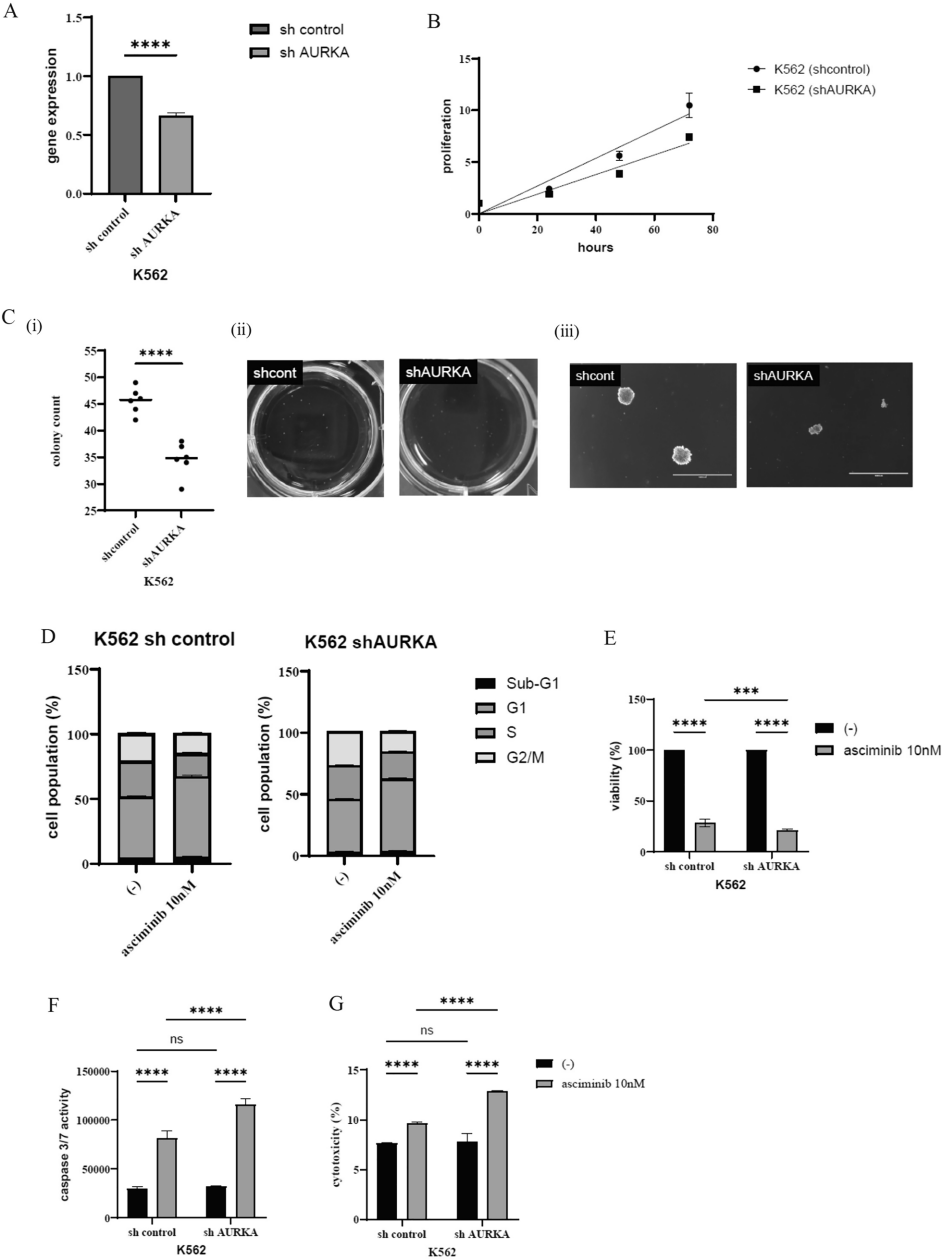
Analysis of the cell proliferation by AURKA shRNA transfection. **A** Gene expression of AURKA was evaluated using a real-time PCR analysis. Significance is expressed as ****p < 0.0001 vs. the control shRNA transfectant cells. **B** Cellular proliferation of shRNA-transfected K562 cells was evaluated with a cell counting kit-8. **C** shRNA-transfected K562 cells (1 × 10^2^ cells) were plated in triplicate on dishes containing a methylcellulose medium. The colony numbers were calculated. The number of colonies detected for 7 days (i), photographs of colonies taken using a digital camera (ii), and a magnified image (4X) of a colony on EVOS™ FL Digital Inverted Fluorescence Microscope (iii). Significance is expressed as ****p < 0.0001 vs. the control shRNA transfectant cells. **D** shRNA-transfected K562 cells were treated with asciminib for 24 h. Cell cycle phase profiling was determined using a BD Cycletest™ Plus DNA Reagent Kit. A representative histogram for each condition is illustrated. **E, F, G** shRNA-transfected transfected K562 cells were treated with asciminib for 48 or 72 h. Cell viability (**E**), caspase 3/7 activity (**F**), and cytotoxicity (**G**) were evaluated (***p < 0.001, ****p < 0.0001, and ns: not significant vs. the control)

This study aimed to investigate the function of AURKA by employing a widely used approach for stable gene knockdown, short hairpin RNA (shRNA)-mediated gene silencing. Subsequently, the effects of AURKA knockdown on CML cells were examined. K562 cells were stably transfected with expression vectors encoding shRNAs targeting AURKA or non-targeting shRNA using a standard lentiviral construct. The efficiency of gene silencing was confirmed using RT-PCR (Fig. 5A). To compare cell proliferation, cells were incubated at a final concentration of 1 × 10^5^ cells/mL. The results indicate that cell proliferation was significantly reduced in the AURKA shRNA-transfected K562 cells compared with the control shRNA-transfected cells (Fig. 5B). Furthermore, it was found that the colony count was reduced in the AURKA shRNA-transfectant K562 cells compared with the shControl transfected K562 cells (Fig. 5C). A cell cycle analysis via flow cytometry was conducted, and the results show that AURKA shRNA transfected K562 cells had an increased G2/M cell cycle population (Fig. 5D). Next, the effectiveness of asciminib in shRNA-transfected K562 cells was evaluated. The results demonstrate that cell viability was notably reduced in response to the asciminib treatment in the AURKA shRNA-transfected K562 cells compared with the control shRNA-transfected cells (Fig. 5E). Additionally, caspase 3/7 activity and cytotoxicity were increased by asciminib in the AURKA shRNA-transfected cells (Fig. 5F, G). These findings suggest that AURKA knockdown enhances the efficacy of asciminib in CML cells.

## Discussion

This study discovered the anti-leukemia effects of asciminib and LY3295668 in ABL TKI-resistant CML cells. Combining asciminib and LY3295668 might be a strategic approach to overcome ABL TKI-resistant cells. Employing a combination of asciminib and LY3295668, which possess distinct mechanisms of action, might make it more challenging for resistant cells to evade both drugs simultaneously. By utilizing the GEO database, it was confirmed that AURKA and AURKB expressions are elevated in BC CML patients. Therefore, targeting AURKA and AURKB may be a promising strategy for advanced CML and inhibitor-resistant CML.

This study demonstrated that the AURKA inhibitor LY3295668 is effective against ABL TKI-resistant CML strains. Hence, AURKA inhibitors, including LY3295668, have shown great potential for enhancing the efficacy of multiple established therapeutic agents in CML cells. It is worth noting that AURKA is also known as an oncoprotein [9, 10]. These results indicate that treating K562 cells with shRNA AURKA reduced AURKA expression and made Ph-positive cells more sensitive to the cytotoxic and apoptotic effects of asciminib. Combining multiple drugs with different mechanisms of action may help overcome resistance by targeting multiple pathways involved in leukemia growth and survival simultaneously.

The activity of β-galactosidase is a hallmark of cellular senescence [18]. Cellular senescence is a stable state of cell cycle arrest that is triggered by stress or damage, such as the inhibition of AURKA. ROS are highly reactive molecules that are naturally produced in cells as byproducts of normal cellular metabolism [19]. Cancer treatments, including those for leukemia, can promote apoptosis, specifically in cancer cells, by increasing ROS activity. The findings of this study indicate that asciminib and LY3295668 induce a senescence-like phenotype in CML cells, characterized by elevated β-galactosidase activity and increased production of ROS. By upregulating ROS activity, cancer treatments, including leukemia, may promote apoptosis, specifically in cancer cells. These results also indicate that asciminib and LY3295668 induced a senescence-like phenotype in CML cells, including elevated β-galactosidase activity, accompanied by increased production of ROS.

Advanced therapies, such as TKIs, have improved the prognosis for individuals with CML. With these treatments, the survival rate for those with the disease is now comparable to that of healthy individuals [1]. Effective monitoring of leukemia is crucial for optimizing treatment outcomes, improving quality of life, and increasing the chances of long-term survival for patients with CML [20]. However, in some cases, point mutations in BCR::ABL1 are the primary cause of acquired TKI resistance in CML. Furthermore, eliminating TKI resistance is the most effective therapeutic approach for reducing the leukemic disease burden and potentially achieving a cure [21]. As a result, the co-treatment of asciminib and LY3295668 significantly decreased the number of CML cell colonies, including those resistant to TKIs, and may be effective in targeting residual CML cells in the bone marrow.

Aurora kinases have garnered significant attention due to their potential to improve the effectiveness of established therapeutic agents across a range of preclinical and clinical trials. LY3295668, a selective inhibitor of AURKA, has demonstrated a high level of selectivity, with over 1000-fold greater potency against AURKA than AURKB. In addition, LY3295668 has shown anti-tumor activity in various cancer cell lines and animal models [22]. Encouragingly, LY3295668 has also exhibited anti-tumor activity in patients with advanced or metastatic solid tumors, with a tolerable toxicity profile in phase I clinical trials [22]. In the phase I study, plasma concentrations of LY3295668 from 13 patients were analyzed [23]. The concentration of LY3295668 in this study was deemed adequate for conducting pharmacokinetic analysis. Several high-specificity inhibitors for AURKA have been created, and several of these have demonstrated clinical effectiveness in clinical trials. In mantle cell lymphoma and upper gastrointestinal adenocarcinomas, combining AURKA inhibitors with docetaxel has been shown to produce better treatment outcomes than using docetaxel alone [8]. Research has revealed that the HDAC inhibitor vorinostat synergistically enhances the lethality of MK-0457 in leukemia and breast cancer cells [24].

For individuals with the T315I BCR::ABL1 gene mutation, ponatinib and asciminib could be viable treatment options. However, achieving success in this context often requires higher doses of the medication [25]. This study demonstrated that lower concentrations of asciminib and LY3295668 effectively induced cell death in T315I mutant, nilotinib-resistant, and ponatinib-resistant CML cells. It also was observed that asciminib and LY3295668 inhibited the growth of CML cells, including those with ABL TKI resistance. According to these results, ABL TKI-resistant cells are more sensitive to asciminib and LY3295668 co-treatment.

Based on this study, the STAMP inhibitor asciminib may be beneficial for patients who have developed resistance to ABL TKIs, and combining this inhibitor with pharmacological aurora kinase inhibition could further improve its effectiveness. Resistance to ABL TKIs in the treatment of CML presents a significant obstacle. It is crucial to emphasize the significance of this study for this patient population, as treatment options for individuals who have become resistant to the available TKIs are currently very limited. Therefore, the potential for asciminib combined with LY3295668 to improve the survival of CML patients, particularly those resistant to ABL TKIs and have experienced treatment failure, is noteworthy.

## Data Availability

The datasets generated and/or analyzed during the current study are available from the corresponding author on reasonable request.
